# Multi-Response Optimization of Processing Parameters for Micro-Pockets on Alumina Bioceramic Using Rotary Ultrasonic Machining

**DOI:** 10.3390/ma13235343

**Published:** 2020-11-25

**Authors:** Basem M. A. Abdo, Hisham Alkhalefah, Khaja Moiduddin, Mustufa Haider Abidi

**Affiliations:** Advanced Manufacturing Institute, King Saud University, Riyadh 11421, Saudi Arabia; halkhalefah@ksu.edu.sa (H.A.); khussain1@ksu.edu.sa (K.M.); mabidi@ksu.edu.sa (M.H.A.)

**Keywords:** RUM, alumina, surface roughness, surface morphology, micro-pockets, edge chipping, MRR, optimization

## Abstract

The machining of ceramic materials is challenging and often impossible to realize with conventional machining tools. In various manufacturing applications, rotary ultrasonic milling (RUM) shows strengths, in particular for the development of high-quality micro-features in ceramic materials. The main variables that influence the performance and price of the product are surface roughness, edge chipping (EC), and material removal rate (MRR) during the processing of ceramics. RUM has been considered in this research for the milling of micro-pockets in bioceramic alumina (Al_2_O_3_). Response surface methodology in the context of a central composite design (CCD) is being used to plan the experiments. The impacts of important RUM input parameters concerning cutting speed, feed rate, depth of cut, frequency, and amplitude have been explored on the surface roughness in terms of arithmetic mean value (Ra), the EC, and the MRR of the machined pockets. The main effect and the interaction effect of the implemented RUM parameters show that by providing a lower feed rate and cutting depth levels and elevated frequency and cutting speed, the Ra and the EC can be minimized. At greater levels of feed rate and cutting depth, higher MRR can be obtained. The influence of RUM input parameters on the surface morphology was also recorded and analyzed using scanning electron microscopic (SEM) images. The study of the energy dispersive spectroscopy (EDS) shows that there is no modification in the alumina bioceramic material. Additionally, a multi-response optimization method has been applied by employing a desirability approach with the core objectives of minimizing the EC and Ra and maximizing the MRR of the milled pockets. The obtained experimental values for Ra, EC, and MRR at an optimized parametric setting were 0.301 µm, 12.45 µm, and 0.873 mm^3^/min respectively with a combined desirability index value of 0.73.

## 1. Introduction

The applications of advanced ceramics are increasing day by day. For example, previously turbine blades and nozzles were only produced by using the super-alloys. However, ceramics are now steadily supplanting super-alloys for the production of turbine blades and other engine and turbine components due to substantial advances in production methods [1]. In addition, in contrast to other similar materials such as super-alloys (Ti-6Al-4V and Inconel 718), ceramics also bring significantly high strength to weight ratio, anti-corrosive characteristics, and increased thermal stability. The applications of ceramics are also increasing in the manufacturing of precision components such as micro-dies, micro-molds, micro-nozzles, and micro-fluidics [1]. In miniaturized/micro-components, micro-features such as micro-pockets and microchannels with robust surface quality are often needed for their deployment with ever-increasingly stringent guidelines [2]. Alumina (Al_2_O_3_) is among the ceramic materials most widely used in biomedical microfluidic applications because of its high adherence to biocompatibility requirements [3]. The material is known to be challenging to cut because of its high strength and non-conductivity (electrical and thermal) [4].

Different technologies had been applied in the previous studies to machine Al_2_O_3_ including laser ablation process [5,6,7], electric discharge machining [8], abrasive water jet machining [9], and grinding process [10]. None of them, however, seemed to be a comprehensive solution with economical machining cost in terms of process quality aspects (low tool wear, minimum edge chipping, minimum surface roughness, high MRR, and high dimensional accuracy). For example, the conventional grinding process has the limitations of higher cutting forces and high edge chipping [11]. Laser beam machining (LBM) application is constrained because of the inadequate surface finish of the machined component and the formation of the recast layer and ‘heat-affected zone’ (region neighboring to the machining zone where structural characteristics alter due to microstructure changes as a result of extreme heat generation), that also has a negative impact on the service life of the product [12,13]. For example, the LBM has been performed to mill pocket on Al_2_O_3_ [5]. The study found that, under certain laser input conditions, the minimum surface roughness of the machined pockets was 3 μm. Microchannels of varying sizes were produced using the laser beam process on Al_2_O_3_ and ZrO_2_ [7,14,15]. The authors addressed the significance of laser beam parameters for channel surface roughness and geometrical accuracy. Research has shown that surface roughness and geometrical precision can be controlled by adjusting the laser process input parameters. The performance of electrochemical machining (ECM) is reliant on the work material’s chemical affinity. Some other ECM disadvantages are low power efficiency and substandard surface quality [14]. Electrical discharge machining (EDM), which is capable of machining only conductive materials, is not practical for ceramic material machining. The EDM output is further impeded by poor MRR, reduced energy efficiency, and high tool wear [15]. In addition, the key disadvantages identified during the machining of alumina materials have been the decreased material removal rate and limited dimensional precision [16]. Consequently, in order to address the quantitative and qualitative technological attributes of the production industry, there is a need to resolve the major weaknesses of the mentioned methods.

Rotary ultrasonic milling (RUM) is among the newest viable production technology used to address the machining challenges of advanced ceramics, such as Al_2_O_3_. It is an entirely mechanical process that can transform conductive and non-conductive materials without altering the machined surface’s microstructural and mechanical characteristics [17,18]. The key benefit of RUM is its ability to manufacture high precision high surface quality of the components and tool life extension [19,20]. It can be considered for both brittle and difficult to process materials for micro/macro machining [21,22,23]. RUM has lately been used in Al_2_O_3_ materials for milling micro-channels and drilling micro-holes. For example, RUM was employed by Abdo et al. [24] to produce microchannels on Al_2_O_3_. The authors investigated the effect of RUM input parameters on the fabricated micro channel’s dimensional accuracy, edge chipping, and surface roughness. The findings demonstrated that the higher dimensional accuracy (width and depth error ≥ 5%) of the machined microchannels with smooth surface roughness (Ra = 0.27 μm) can be attained under optimal RUM parameter settings. Another attempt has been performed by Abdo et al. [25] in which RUM was applied for milling microchannels in pure alumina. Results showed that smooth surface morphology along with low surface roughness (Ra = 0.21 µm) of the machined surface of alumina can be obtained at certain levels of RUM input parameters. In order to investigate the effects of the variables on the cutting force, MRR, and chip size of the drilled holes, RUM was also exploited for drilling Al_2_O_3_ [26,27]. The objectives explored during the side milling of Al_2_O_3_ by using RUM in the work described in [28] were tool wear and MRR. The analysis of RUM tool wear and surface roughness while grinding Al_2_O_3_ was reported in [29] and [30], respectively. Results showed that the RUM could achieve high MRR with the less cutting force for alumina ceramic.

RUM was also applied for machining different hardtop- cut materials such as glass and titanium alloys. For example, the effects of cutting and vibrating parameters of RUM on the machining quality of titanium alloy (Ti-6Al-4V) have been examined by Ni et al. [31]. The experimental results showed that the cutting force, surface morphology, and chipping of the machined part could be improved by using RUM comparing to conventional milling. The RUM was also used as a finishing process of the additive manufactured parts of titanium alloy [32]. The relationship between the RUM parameters and edge chipping of the holes drilled on float glass has been investigated by Sharma et al. [33]. Results found that the least volume of chipping is estimated as 2.12 mm^3^ at low levels of feed rate, high levels of amplitude, and less tool diameter. Liu et al. [34] used response surface methodology as an optimization tool to optimize RUM parameters in order to minimize the edge chipping and tool wear while drilling holes in alumina materials. Results showed that the low feed rate, an adequate amount of amplitude as well as high cutting speed were selected as optimal parameters which result in decreasing the tool wear and exit crack of the drilled holes as 16.891 μm and 25.375 μm, respectively. Feed rate was found to be the most significant factor affecting the MRR and surface roughness of the holes drilled in BK7 glass using RUM [35].

It can be noticed from the current literature mentioned that few scientific studies on the micro-milling of Al_2_O_3_ have been carried out. In addition, hardly any literature on milling pockets in Al_2_O_3_ using RUM has been published. The literature reviews also stated that no detailed analysis for process optimization of Al_2_O_3_ while RUM was undertaken. Most of the time, the researchers followed a single factor at a time strategy for conducting experimental work that offers the only generalized variability of process responses with input variables but lacks meaningful knowledge regarding the combined influence of all the variables engaged in the analysis. This void explicitly needs statistical methods such as response surface methodology to structure the experimental matrix and multi-response optimization of qualitative features of the method under examination to be used in the design of experiments (DOE). In addition, the majority of studies available were restricted to assessing the influence of RUM input parameters on the cutting force and tool wear of manufactured holes and/or channels. This gap demands the exposure of the effects of RUM parameters on the EC which are the common problems faced while machining brittle materials such as Al_2_O_3_. The effect of the most important vibration parameters of RUM such as vibration frequency and vibration amplitude was neglected and kept constant while applying RUM in machining brittle materials. The most challenging task of obtaining the highest MRR to maintain the surface roughness and EC to their least acceptable levels was also disclosed in the literature. In any machining operation, the assignment of simultaneous optimization of these variables can be a valuable advancement to address the real-life problems of the industrial field.

Based on these research gaps described above, this paper focuses on the performance analysis of RUM while milling of Al_2_O_3_. The impact of RUM input parameters (cutting speed, feed rate, depth of cut, vibration frequency, and vibration amplitude) on the responses (EC, Ra, and MRR) has been investigated. Response surface methodology has been applied to formulate the experimental work and then, the adequacy of existing models is verified by analysis of variance. The desirability approach has been subsequently adopted for individual optimization as well as simultaneous optimization of the responses. In order to shed light on surface morphology and the EDS of the machined surface, SEM analysis has also been performed.

## 2. Material and Method

**Figure 1:**
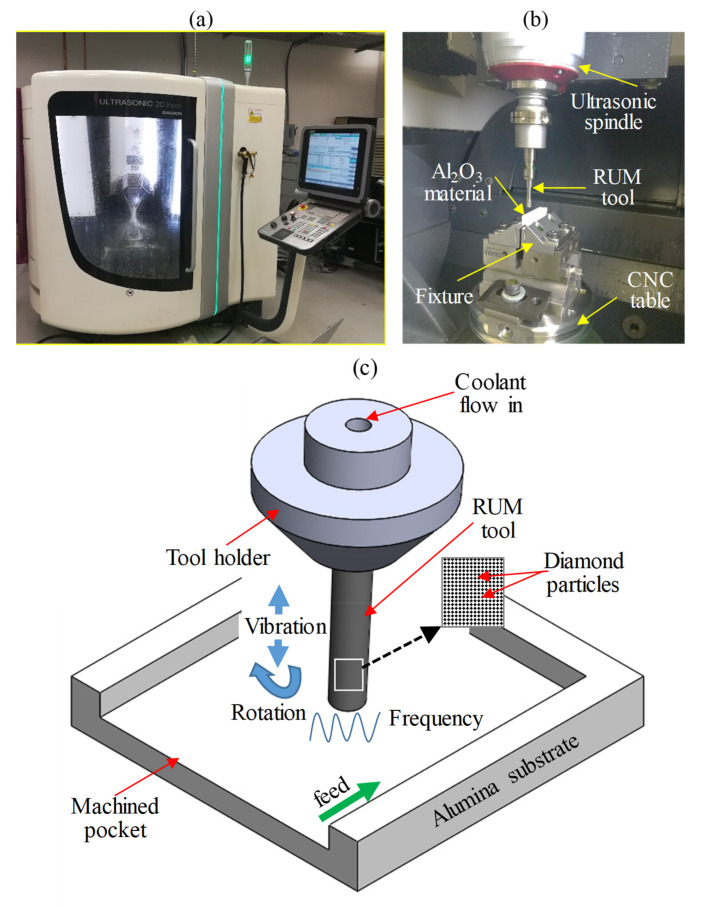
(**a**) RUM machine, (**b**) experimental set up, and (**c**) schematic of RUM.

The workpiece material selected in this investigation was alumina bioceramic from CeramTec (Plochingen, Germany). It is an effective material for the fabrication of microfluidics and lab-on-a-chip device [36,37]. For the efficient functioning of these devices, the micro-features such as microchannels and micro pockets represent the critical components. The higher the precision or the accuracy of these micro-features, the better will be the performance of these devices. Therefore, this work has focused the optimization of RUM parameters that can provide high-quality micro-pockets. The dimensions of alumina samples employed in the present work were 50 × 10 × 10 mm. The characteristics of the alumina material are depicted in Table 1. A DMG ultrasonic 20 linear machine (Geretsried, Germany), as illustrated in Figure 1a was preferred to mill the micro-pockets on the alumina material. It is a five-axis precise machine that is primarily used for machining hard to cut materials especially brittle materials such as ceramics and glasses. The experimental layout can be noticed in Figure 1b and the schematic of RUM used in this study is shown in Figure 1c.

**Figure 2:**
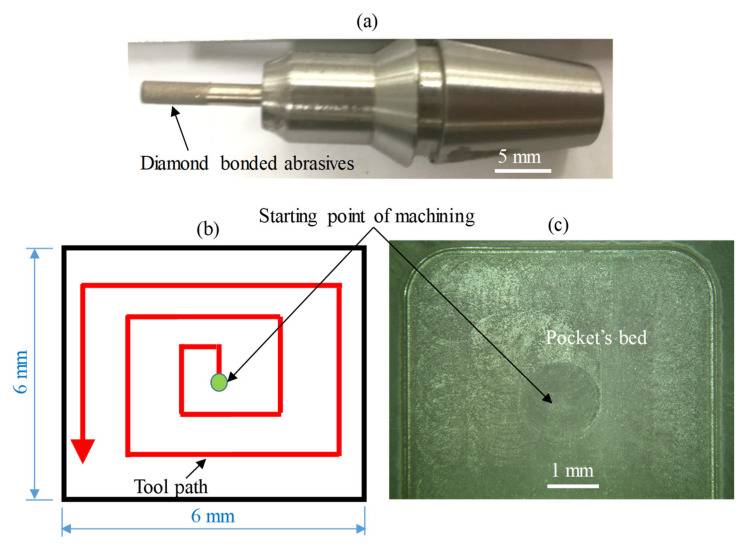
(**a**) RUM used tool, (**b**) schematic of the tool path of the machined pocket, (**c**) a sample of an actual machined pocket.

In the present study as described in Figure 2a, nickel bonded diamond RUM tools with a diameter of 2 mm supplied by Schott company, (Mitterteich, Germany) have been used. The cross-sectional measurements of the micro-pocket adopted in this investigation are 6 mm (length and width) × 0.3 mm (depth). The tool bath followed while machining the pockets is shown in the schematic diagram in Figure 2b. Figure 2c shows a sample of the actual machined pocket. The prior literature reporting the micro-machining of brittle materials using RUM [23,26,34,35] and the results of the preliminary experiments were utilized to choose the appropriate range of RUM parameters. The vibration frequency of the used RUM cutting tool was measured and found that the resonance ranges to be between 20 and 30 kHz. So the three levels of vibration frequency used in this study were 20, 25, and 30 kHz. Note that the vibration frequency produced by the piezoelectric transducers of the used DMG ultrasonic spindle ranges between 17.5 and 48 kHz. Regarding the amplitude, it is a common practice in the RUM to express the vibration amplitude as a percentage of the ultrasonic power [21,39,40]. However, in this study, the relationship between the ultrasonic power and the vibration amplitude has been taken from the previous literature by Abdo et al. [25].

To investigate EC, Ra, and MRR in RUM of Al_2_O_3_ bioceramic, five input process parameters—namely cutting speed, feed rate, depth of cut, vibration frequency, and vibration amplitude—were selected. By altering the chosen parameters in their defined range, the various sets of experimental conditions were achieved, while other machining conditions or variables were unaltered throughout the experimental task. Table 2 depicts the RUM input parameters with their corresponding levels and the fixed parameters applied in the experiments.

The experiments were planned and designed through CCD in the current study utilizing an exploratory technique termed ‘response surface methodology’ (RSM). Minitab V19 software (Minitab Inc., State College, PA, USA) was adopted for this reason. The advantages offered by the RSM is to determine the interaction between the independent variables, modeling the system mathematically, and saving time and cost by reducing the number of trials [41]. In the experimental design, there had been five process parameters, each having three levels. According to the experimental design, all 32 trials were carried out in a completely randomized fashion to eliminate experimental errors. Micro-pockets in alumina ceramic workpieces were milled under various operating conditions depending on the planned experimental matrix.

**Figure 3:**
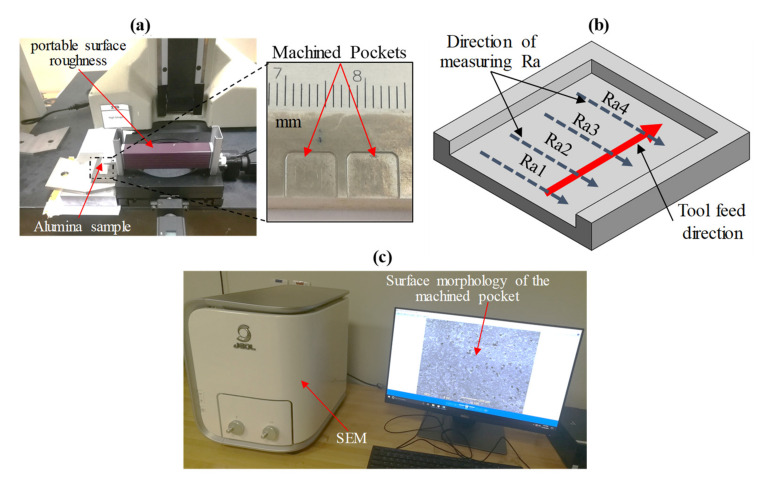
(**a**) Set up of measuring Ra, (**b**) schematic of measuring the surface roughness (Ra), and (**c**) surface morphology measuring set up.

Surface roughness (Ra) was evaluated at four spots (Ra1, Ra2, Ra3, and Ra4) across the tool feed direction of the tool utilizing a portable surface roughness instrument from Mitutoyo Corporation, Kawasaki, Japan (see Figure 3a,b). For assessment, the average of the four readings was chosen. A tabletop SEM from Jeol, Tokyo, Japan (Model JCM 6000Plus) as presented in Figure 3c, was deployed to assess the exit edge chipping and the surface morphology of the pockets. In any machining process, the MRR is often perceived to be a critical response because it is closely linked to economic output. The MRR has been calculated as

(1) $$\mathrm{MRR}=\frac{V}{t}$$

where *V* is the volume of the machined pocket and t is the machining time. The volume is constant of all experiments (volume = width × length × depth) of the machined pocket; (*V* = 6 × 6 × 0.3 mm = 10.8 mm^3^). The machining time, which depends on feed rate and depth of cut, has been calculated by the Siemens controller (Munich, Germany) built in the DMG machine (Geretsried, Germany). A CCD based on response surface methodology was preferred to structure the experimental task.

## 3. Results and Analysis

**Figure 4:**
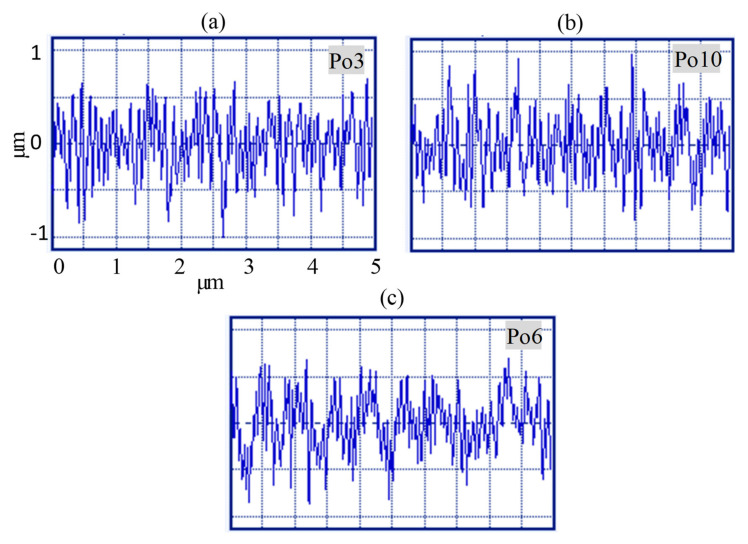
2D surface profiles of the fabricated pockets (**a**), Exp. #1 (**b**) Exp. #14, (**c**) Exp. #21.

Table 3 illustrates the experimental findings of thirty-two micro-pockets machined in alumina (Al_2_O_3_) under numerous RUM process parameters by implementing the CCD. Surface roughness (Ra), EC, and MRR are regarded as the main performance measurements in the rotary ultrasonic milling operation. The Ra and EC values reported for each experiment displayed in Table 3 are the mean of the four readings. Figure 4 highlights the descriptions of the generic scanned 2D profiles of micro-pocket milled at various input parameters. It demonstrates that the profiles of roughness vary from smooth (see Figure 4a) to rough with deep pits (see Figure 4c) and indicates that the RUM process variables significantly impact the roughness of the milled micro-pockets. To analyze the relevance of the developed models, the analysis of variance (ANOVA) test was also conducted. Only the relevant parameters in the models were used. Table 4, Table 5 and Table 6 for Ra, EC, and MRR, respectively, outline the ANOVA test outcomes for the response variables. Depending on the ANOVA test outcomes stated in Table 4, Table 5 and Table 6 the estimates of the probability term (*p* < 0.05) statistically validate the relevance of model terms at the 95% confidence interval. The models with F-values of 12.99, 13.63, and 28,435.72 suggest that they are statistically significant and the findings fit into them fairly adequately. Because the intended setting for the calculation and interpretation of the results to be valid, the lack of fit for the developed model requires to be ‘not significant’. The *p*-value for lack of fit is 0.549, 0.11, and 0.541 for Ra, EC, and MRR for all models, which establishes this as an irrelevant term for pure error. The ratio of the term ‘sum of squares’ for individual sources to the ‘total of sum of squares’ was also determined as the percentage contribution for all models. For Ra, EC, and MRR, the percentage effect of ‘pure error’ is 0.7%, 0.03%, and 0.001%, respectively. For all models, this smaller value of pure error means that there is an almost marginal difference in the experimental data induced by error, and thus, in the present study, the variation through the chosen design parameters supersedes the variation through error. This further verifies the ANOVA analysis and its outcomes. Another imperative coefficient in the ANOVA analysis is ‘R^2^’, which explains the amount of variance expressed by the model concerning the total variability in the actual data [26]. In Table 4, Table 5 and Table 6, the calculated values of 0.9828 and 0.9259 indicate that the model shows 94.45%, 92.54%, and 99.9% variability of Ra, EC, and MRR, respectively.

Based on the RSM approach, the developed models for Ra, EC, and MRR are represented in Equations (2)–(4), after employing the method of ‘backward elimination’ for obliterating ‘not significant’ parameters.

(2) $$\begin{array}{ll} Ra=\;0.607\;- & 0.00000001\times \;A-\;0.0038\;\times B\;+\;0.711\;\times C\;-\;0.01602\;\times D\\ & -\;0.01641\;\times E\;-\;0.000001\;\times A\times D\;+\;0.000002\;\times A\times E\;\\ & +\;0.00325\times \;B\times D\;-\;0.00575\;\times B\times E\;+\;0.0008\;\times D\times E \end{array}$$

(3) $$\begin{array}{ll} EC=16.73- & 0.001813\times A+0.808\times B+30.3\times C-0.4689\times D+0.621\times E\\ & \;-0.0047\times A\times C+0.000081\times A\times D+8.25\times B\times C\\ & \;-0.11\times B\times E-1.7\times C\times E \end{array}$$

(4) $$\begin{array}{l} MRR=-0.03118+0.0000001\;\times A\;+\;0.00587\;\times B\;+\;0.3660\;\times C\;\\ \;+\;0.000508\;\times D\;+\;0.001310\;\times E\;-\;1.422\;\times C^{2}\\ \;-\;0.000001\;\times A\times B\;-\;0.000010\;\times A\times C+\;0.0000001\;\times A\times D\;\\ \;+\;1.39633\;\times B\times C\;+\;0.000385\;\times B\times E\;+\;0.00962\;\times C\times E\;\\ \;-\;0.000116\;\times D\times E \end{array}$$

**Figure 5:**
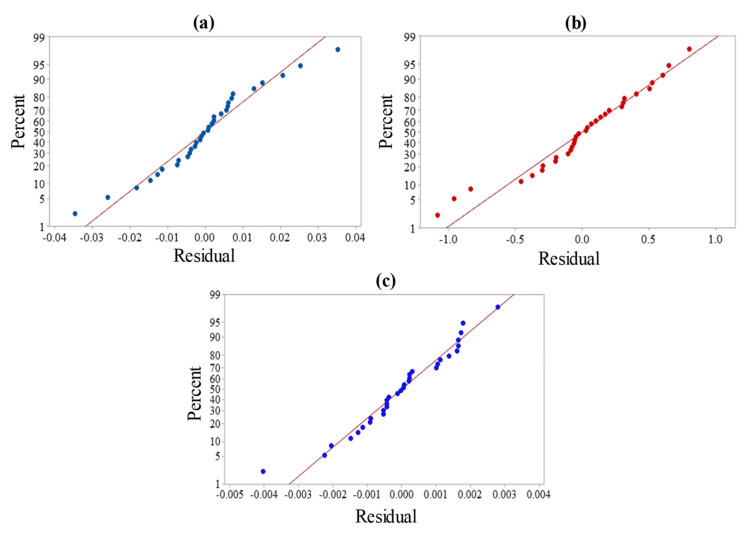
Normal probability plots for (**a**) Ra, (**b**) EC, and (**c**) MRR.

**Figure 6:**
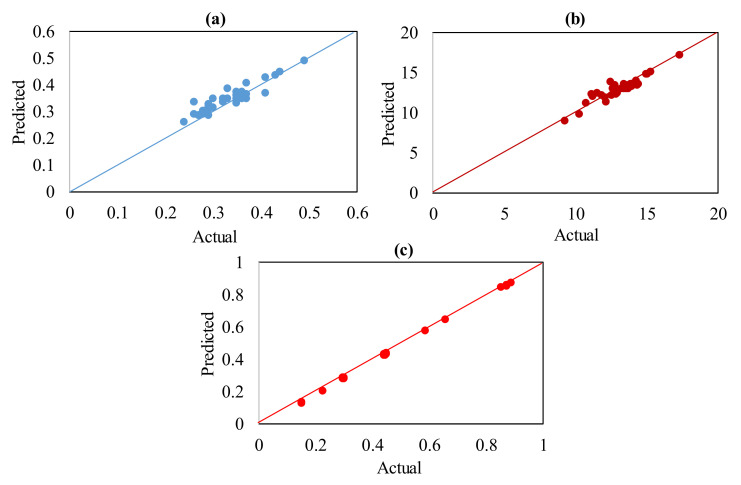
Actual v/s predicted plots for (**a**) Ra, (**b**) EC, and (**c**) MRR.

The normal probability plots for Ra, EC, and MRR respectively, are displayed in Figure 5a–c. These plots highlight that the majority of the residuals are distributed out along the best-fitted line, further demonstrating the normal distribution of the errors. The verification of the built models is carried out by evaluating real values with predicted values. Figure 6a–c displays the measured values for the explored responses—i.e., Ra, EC, and MRR, respectively—against the expected values plots. The established regression models are consistent with the actual values, as interpreted from these plots. The prediction made for interpreted responses (i.e., Ra, EC, and MRR) with the established regression models is thus substantiated with precision and reliability.

**Figure 7:**
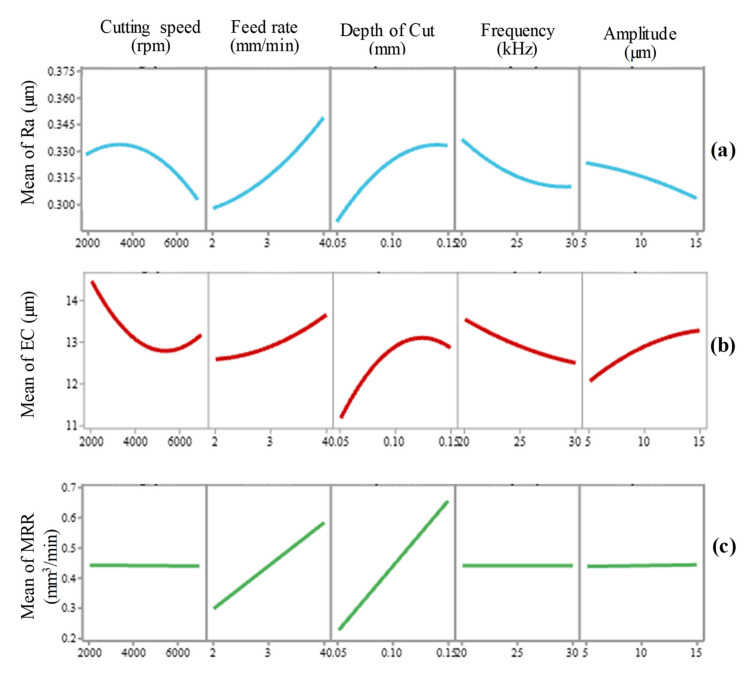
Main effect plots of (**a**) Ra, (**b**) EC, and (**c**) MRR.

The main effects of the factors (see Figure 7), explicitly cutting speed (A), feed rate (B), cut depth (C), frequency (D), amplitude (E), are reported to have major effects on Ra and EC in the RUM of alumina ceramic. The MRR was, though, found to be impacted only by the feed rate and cutting depth.

Figure 7a–c demonstrates the trends in machined pocket responses (Ra, EC, and MRR) with the alterations of RUM input parameters using the main effect plots. As feed rate and depth of cut levels raised from 2 mm/min to 4 mm/min and 0.05 mm to 0.15 mm, respectively, Ra is noticed to be sharply gone up, as seen in Figure 7a. Higher levels of cutting speed, frequency, and amplitude lead to a sharp decline in Ra. With an increase in feed rate, depth of cut, and amplitude, EC is discovered to rise as shown in Figure 7b. EC, on the other side, reduces with enhanced cutting speed and frequency (see Figure 7b). In terms of the main effect, only feed rate and depth of cut are observed to possess significant influences on MRR as illustrated in Figure 7c. MRR increases with a rise in feed rate and depth of cut.

**Figure 8:**
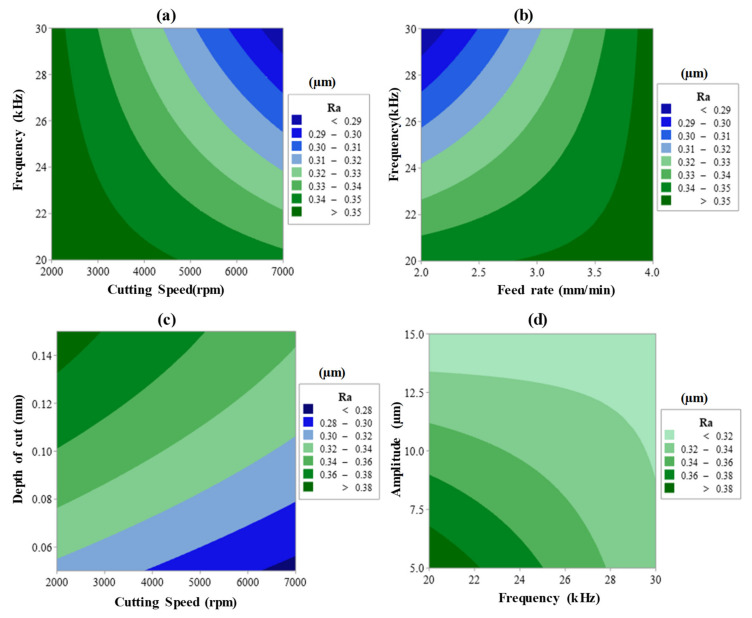
Two variable interactive effects of (**a**) cutting speed × frequency, (**b**) feed rate × frequency, (**c**) cutting speed × depth of cut, and (**d**) frequency × amplitude on Ra.

**Figure 9:**
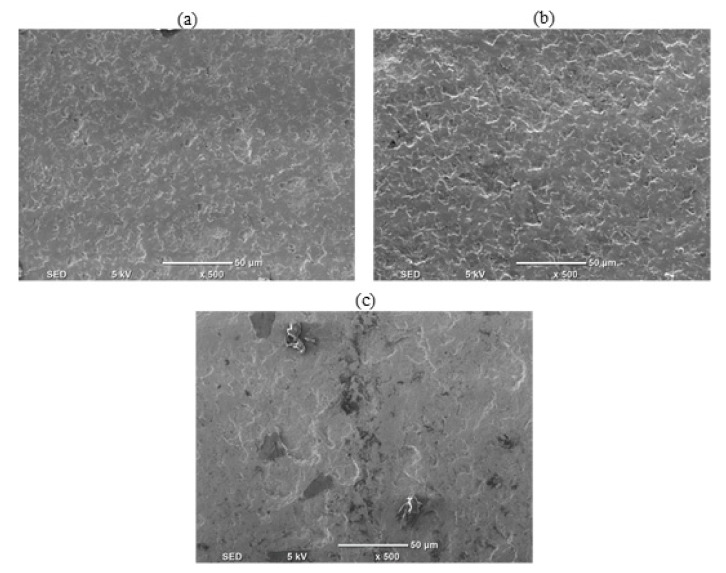
Surface morphology of the machined pockets (**a**) Exp. #3, (**b**) Exp. #10, and (**c**) Exp. #6.

The interaction effect was also found to have a significant effect on all responses. For example, Ra and EC affected by cutting speed × frequency (A × D) and feed rate × amplitude (A × E). MRR is affected by the interaction of feed rate and depth of cut (B × C). Concerning the second-order term, only the depth of cut was reported to have an impact on the MRR. All contribution percentages of the main effects, interaction effects, and second-order effects were illustrated in Table 4, Table 5 and Table 6. Surface plots are exemplified with the combined effects of input process parameters on Ra, as presented in Figure 8a–d. Whereas Figure 9a–c introduces the SEM images showing the impact of the RUM input variables on the machined surface morphology as noticed on the pocket bed. The surface plots for the outputs are mentioned only against the most important RUM parameters for the sake of clarity as shown earlier in Table 4, Table 5 and Table 6. The researchers address surface roughness to understand the impact of the RUM process factors on the machinability of alumina material (Figure 8) and surface morphology (Figure 9) since both are closely related to each other. The aggregate effect of cutting speed and frequency on Ra is shown in Figure 8a. It can be demonstrated that the surface roughness decreases with increasing cutting speed and frequency. As the cutting speed surged from 2000 to 7000 rpm, Ra decreased from 0.49 to 0.24 μm with a 51% reduction. The value of Ra improved from > 29 μm to > 0.40 μm when the feed rate raised from 2 to 4 mm/min, as noticed in Figure 8b. As the cutting speed increases, the exposure time of the machined surface and the diamond abrasives on the RUM tool also increases. That is the same area on the machined surface is repeatedly abraded/scuffed by the abrasives and it also contributes to a decrease in the true cutting depth per diamond abrasive and a decrease in the cutting force [42]. This leads to a greater cutting action (plastic removal) and reduction of the ceramic material’s brittle fracture and a reasonably smoother surface is created, as illustrated in the SEM micrograph in Figure 9a. On the other hand, if the feed rate continues to rise, the exertive load from the tool along with the feed direction leads to the knocking out of the machined surface of the pre-cracked materials, creating a rougher surface with deep grooves and pits, as can be observed in Figure 9b. As shown in Figure 8c, Ra has been observed to increase with greater cutting depth. This is again due to a rise in the cutting load per abrasive diamond [32], which contributes to more fracturing and deeper pit creation on the machined surface. It is noticeable in Figure 9c where deeper pits and fragmented surfaces are formed at a greater depth of cut. The cumulative influence of vibration frequency and amplitude on Ra is depicted in Figure 8d. Ra was observed to decline with a rise in both frequency and amplitude. It is because as the amplitude rises, the hammering action of the tool on the machined surface increases, and accordingly pre-cracking in the material also increases, which produces a smooth surface. At higher frequency the crack density increases owing to the more frequent hammering of the tool and thus crack coalescence grows, decreasing crack propagation and contributing to reduced Ra [24].

**Figure 10:**
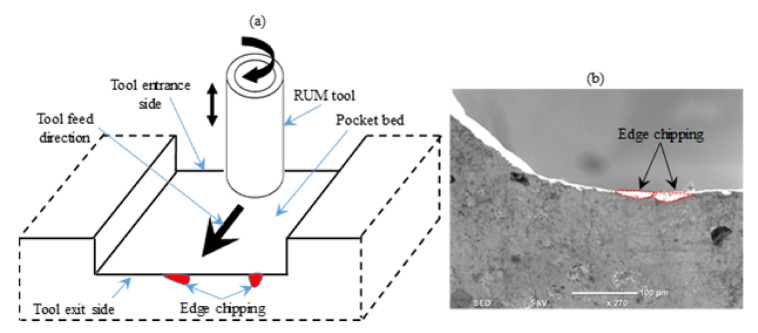
Edge chipping analysis, (**a**) illustration of the chipping spots, (**b**) an example of an outlet edge chipping.

It must be noted that examining the surface roughness on the bed of the pocket is indeed not sufficient to evaluate the quality of the machined pockets. It is because the chipping of the edges is mentioned to occur during the RUM of brittle materials [27]. Edge chipping is one of the key restrictions of the RUM method for the realization of high-quality surfaces on hard and brittle ceramic materials like alumina ceramic [43]. The edge chipping along the tool exit side was explored to further examine the quality of the machined micro-pockets (see Figure 10a). When the tool pulls away from the workpiece, the edge chipping as shown in Figure 8a decreases the strength of the machined material and increases the probability of failure, in addition to influencing the geometric precision of the part [27]. Figure 10b presents an example of the edge chipping along the tool exit side.

**Figure 11:**
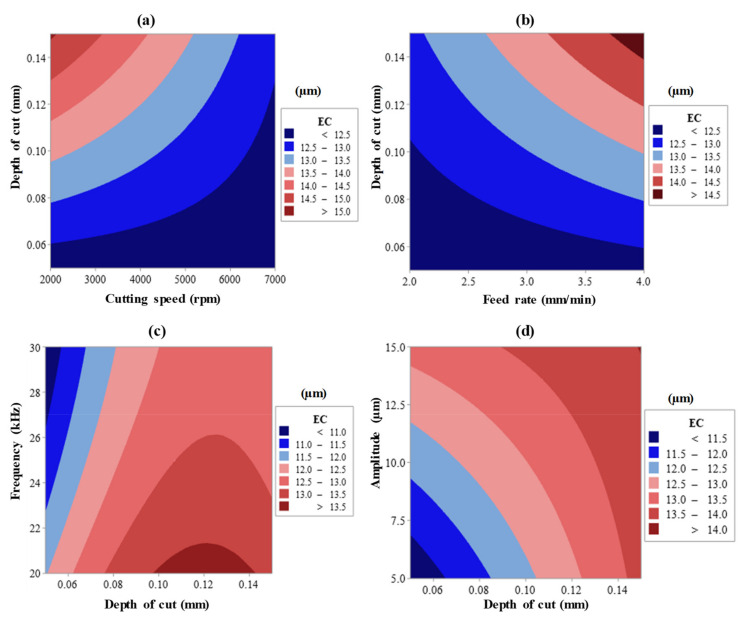
Two variable interactive effects of (**a**) cutting speed × depth of cut, (**b**) feed rate × depth of cut, (**c**) depth of cut × frequency, and (**d**) depth of cut × amplitude on EC.

The cumulative impact of input variables on EC is well-explained using surface plots, as evidenced in Figure 11a–d. For example, it is very well documented in the literature that as the speed of the spindle rises, the cutting force and the feed rate reduces [43]. The overall influence of the cutting speed and depth of cut, power, and feed rate on EC is shown in Figure 9a. It is apparent from Figure 11a that as the cutting speed rises and the cutting depth declines, the chipping of the exit edge dramatically decreases. As the degree of cutting speed rises from 2000 to 7000 rpm, the diamond abrasive penetration depth decreases, but the contact length increases [1,25]. This leads to a decrease in the cutting force at elevated spindle speed. Lower edge chipping at high cutting has therefore been noted. Similarly, the cutting force rises again as the cutting depth increases [44], and the exit edge chipping also rises, as seen in Figure 11b. As for the ultrasonic factors, i.e., frequency and amplitude, the exit edge chipping decreases as the frequency increases, as indicated in Figure 11c. As the amplitude increases (from 5 μm to 15 μm), the EC is observed to rise due to the ramp-up in the hammering action of the tool, which increases the cutting power, as depicted in Figure 11d.

**Figure 12:**
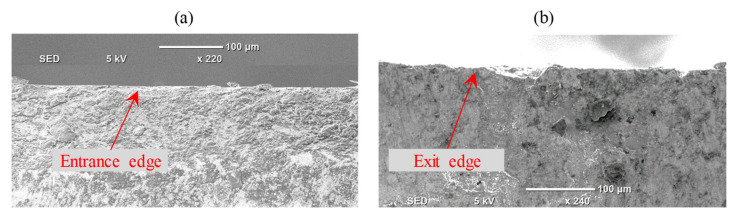
A comparison between the entrance and exit edge chipping (**a**) entrance edge chipping, (**b**) exit edge chipping.

In relation to the chipping of the exit edge, it should be noticed that the chipping of the entrance edge was found to be insignificant. The relation between entrance chipping and exit edge chipping is shown in Figure 12, and it can be verified that the chipping of the entrance edge (refer to Figure 12a) is unnoticeable as opposed to the chipping of the exit edge (view Figure 12b). It is because as the tool reaches the workpiece, it expands compressive forces on the edges of the workpiece and the ceramics are commonly recognized for their exemplary compressive load strength [45]. On the contrary, it produces tensile forces on the outer edge as the tool leaves the workpiece and the ceramics are well renowned for their unfavorable compliance and susceptibility to tensile forces [45]. It is also evident from the mechanical properties of Al_2_O_3_ materials that the maximum tensile strength (650 MPa) is too weak in contrast to the maximum compressive strength (5500 MPa).

**Figure 13:**
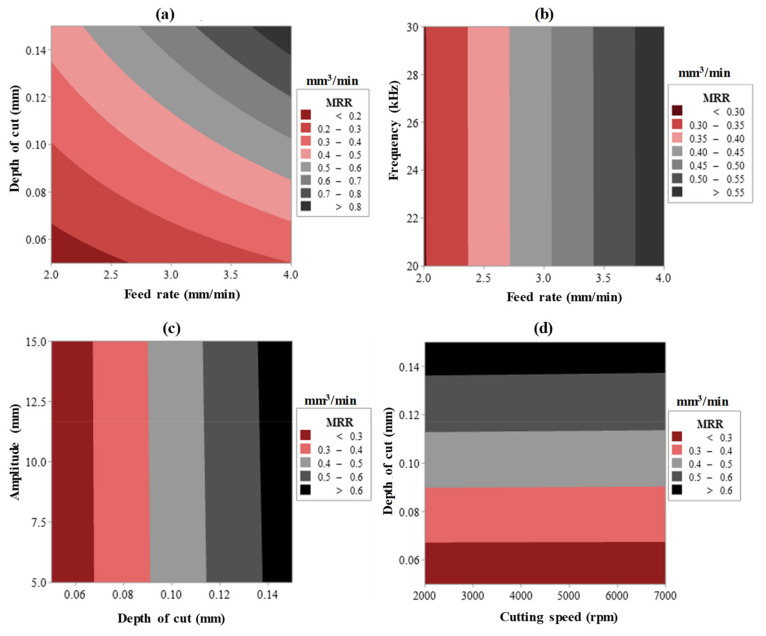
Two variable interactive effects of (**a**) feed rate × depth of cut, (**b**) feed rate × frequency, (**c**) depth of cut × amplitude, and (**d**) cutting speed × depth of cut on MRR.

**Figure 14:**
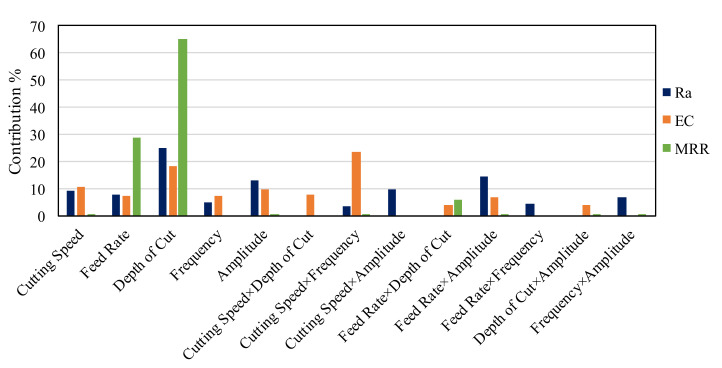
Contribution % of RUM input parameters on Ra, EC, and MRR.

The cumulative impact of the RUM input process variables on the MRR is described utilizing surface plots, as demonstrated in Figure 13a–d. The overall effect of feed rate and depth of cut on MRR is shown in Figure 13a. The increased MRR is noticed as the feed rate of the diamond milling tool rises. The increase in feed rate leads to higher indentation depth of the diamond abrasives bonded on the RUM tool, hence enhanced MRR was detected. The fusion of feed rate and frequency (Figure 13b), depth of cut and amplitude (Figure 13c), and cutting speed and depth of cut (Figure 13c) reveal that only depth of cut and feed rate have a profound influence on MRR. The residual input factors such as cutting speed, frequency, and amplitude possess no impact on MRR. Figure 14 describes the contribution (%) of the main effect and the interaction effect of RUM input variables on Ra, EC, and MRR.

**Figure 15:**
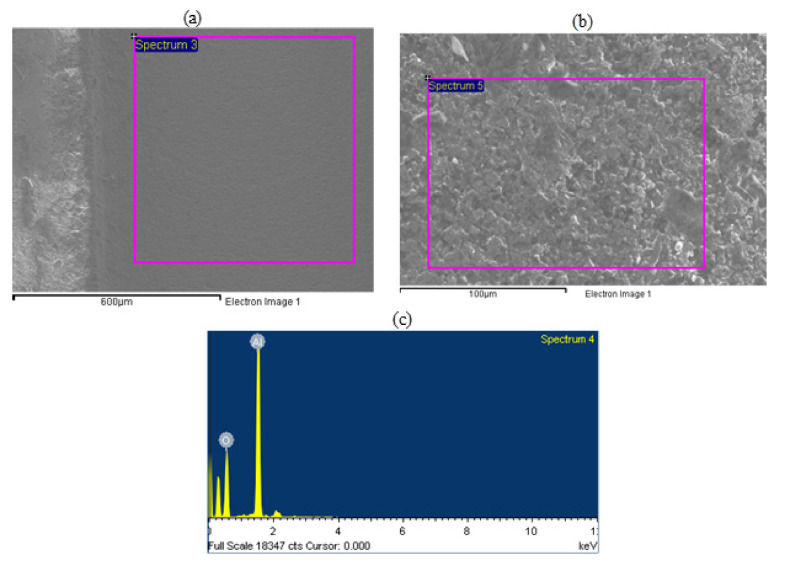
EDS analysis, (**a**) EDS at the pocket bed, (**b**) EDS at pocket side, (**c**) elemental analysis of as-received alumina material.

The elemental composition of the machined pockets was also assessed by utilizing the EDS analysis. The EDS analysis was performed on the pocket beds as well as on the pocket’s sidewalls as depicted in Figure 15a,b respectively. Table 7 presents the comparison of the elemental composition within the micro-pockets and as-received alumina forte material. No modification in the composition of the Al_2_O_3_ material was observed post-machining and no residues of the nickel or diamond were noticed in the EDS study. This indicates that the RUM process could be utilized effectively for the fabrication of Al_2_O_3_ material for medical applications where degradation from the tool could result in a breakdown of the machined components.

## 4. Optimization Results

The explored input process parameters were also optimized for both single and multiple responses to achieve the minimum Ra and EC and maximum MRR. This optimization study was carried out by employing the ‘desirability approach’.

### 4.1. Single Response Optimization

Single response optimization was undertaken to identify the appropriate combination of input parameters to obtain the optimal value of the interesting response. For this reason, all the input variables were confined in a specific space by placing the constraints as provided in Table 8. In the majority of the industrial applications, the lower values of Ra and EC are required while MRR must be higher. For the ideal solution, the Ra and EC were chosen to be minimized, and MRR was set to maximize as presented in Table 8. The aim was to set a ‘range’ for all the input variables, which emphasizes that variables will be optimized through exploring the region within the lower and upper limits. Input variables and responses have been assigned equal weight and importance. The machining solution having an average desirability value of ‘one’ or ‘closer to one’ is selected as the optimized solution. For each response, the most suitable solution was chosen out of potential solutions, which is simple to confirm the predictions made by the models Ra, EC, and MRR. For their convenient setting, the parametric settings were rounded off by the experimental setup control panel as described in Table 8. The outcomes of single response optimization are displayed in Table 9. At these optimum settings, the confirmatory experimental runs have been carried out using two replications, and the predicted values and average of confirmatory experimental results for Ra, EC, and MRR are also presented in Table 9. The confirmatory results for Ra, EC, and MRR have been noticed to differ from predicted values by 2.3%, 0%, and 0.67%, respectively.

### 4.2. Multi-Response Optimization

**Figure 16:**
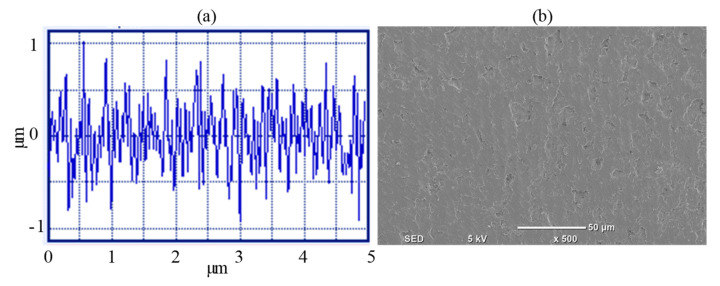
(**a**) The 2D surface profile of the machined pocket at the optimal RUM parameters, (**b**) surface morphology of the machined pocket at the optimal RUM parameters.

The responses—i.e., Ra, EC, and MRR—are exceedingly inconsistent in the RUM process. Low Ra with low EC and high MRR cannot be accomplished concurrently for a specific parametric scenario. While Ra and EC are related to a quality aspect, the MRR is related to the quantity aspect of the RUM process. For industrial purposes, machining capabilities that can maximize the different goals must be acquired. To this purpose, the desirability strategy was implemented for multi-response optimization. In other terms, if the Ra is lesser then the MRR will also be smaller, which is not the optimal case. The consistency of the solution is determined in the sense of the closeness of mean to desired target values. The desirability value of 1 will be reached by a good balance of performance response. To achieve a good compromise between the analyzed responses simultaneously, one with optimum desirability is selected from all the optimal solutions. Table 10 outlines the optimized setting of process variables after rounding-up. Cutting speed of 6787 rpm, the feed rate of 3.52 mm/min, depth of cut of 0.13 mm, frequency of 21.8 kHz, and amplitude of 13.7 µm are the optimal experimental condition for multi-response optimization with the composite desirability of 0.73 as demonstrated in Table 10. To assess the errors, validation tests were performed twice at the optimum settings. During the validation checks, errors of 7.3%, 7.2%, and 1.46% were observed at the optimum for Ra, EC, and MRR, respectively. Figure 16a displays the profile of the 2D surface. At optimal parameters, Figure 16b reflects the surface morphology of the machined pocket.

## 5. Discussion

Alumina is an effective material for the fabrication of micro-devices such as microfluidics and lab-on-chip [36,37]. The micro-features such as microchannels and micro pockets represent the critical components for the efficient functioning of these devices. The higher the precision or the accuracy of these micro-features, the better will be the performance of these devices. However, achieving high quality (less surface roughness (Ra) and minimum edge chipping (EC)) along with higher MRR are considered challenging tasks while machining ceramic components. Therefore, this work has focused on the optimization of RUM parameters that can provide high-quality micro-pockets (low Ra and low EC) with higher MRR. From the analysis, it is evident that MRR was highly dependent on feed rates and depth of cut. This is because the machining time decreased with an increase in the feed rate (mm/min) and depth of cut (mm). The increase of feed rate and depth of cut leads to the enhancement in the penetration depth of diamond abrasives which results in producing higher MRR. Other researchers have also reported a similar type of observation. For example, Alkhalefah H. [46] investigated RUM drilling on alumina ceramic and found that the MRR was affected by the feed rate only. Li et al. [47] performed an analysis of ceramic matrix composites and found development in MRR with a higher feed rate. Similarly, the results of the study carried out by Jiao et al. [48] are matched with the outcomes of the current study. For the remaining parameters (cutting speed, amplitude, and frequency), results showed that they have some effects on MRR (see Table 4, Table 5 and Table 6), but their effect is neglectable compared to the significant effect of feed rate and depth of cut on MRR.

The methodology applied in this work can be employed to accomplish optimal parameters of RUM for milling pockets and channels in other ceramic materials such as ZrO_2_, Si_3_N_4_, and SiC. This study can be applied in many fields, including medical and engineering industries. However, due to the high cost of experimentation, the analysis of the tool wear did not consider in this study. This will need detailed investigations in future work.

## 6. Conclusions

This study has been undertaken to examine the impact of the five main input variables of RUM on surface roughness, edge chipping, and MRR of the micro-pockets milled on Al_2_O_3_ material. The desirability method was also applied to optimize multiple responses. The following conclusions can be made.

- Amongst the chosen input variables, the depth of cut has been noticed as the most prominent parameter for all the responses. Increased depth of cut and feed rate gives rise to the superior solution concerning the material removal rate of RUM.
- The vibration frequency and amplitude were identified to have an influential consequence on the surface roughness and the edge chipping of the produced pockets. The surface roughness reduces with rising frequency and amplitude while the edge chipping escalates with growing amplitude and lessens with greater frequency.
- Smaller feed rate and lesser depth of cut and increased cutting speed furnish the desirable Ra and EC outcomes considering the stress produced in the contact region between tool and workpiece declines with decreased feed rate and depth of cut. The parametric fusion of elevated spindle speed, lower depth of cut, and smaller feed rate is noticed as the effectual outcome for producing sleek and polished surface quality with less edge chipping.
- The parametric combination of cutting speed 6787 rpm, feed rate 3.52 mm/min, depth of cut 0.13 mm, frequency 21.8 kHz, and amplitude 13.7 µm can effectively accomplish optimized multiple responses. The confirmatory experiments achieved the values of 0.301 µm, 12.45 µm, and 0.873 mm^3^/min (with the composite desirability of 0.723) for Ra, EC, and MRR obtained respectively using the optimized settings.
- The effectiveness of the multi-response optimization using the desirability approach has been affirmed by the confirmation experiments with an error of less than 5%.

## Figures and Tables

**Table 1:** Mechanical and thermal characteristics of Al_2_O_3_ [38]

Property	Value	Unit
Hardness	1700	HV 0.5
Compressive strength	5500	MPa
Tensile strength	665	MPa
Fracture toughness	5	MPa
Density	3.89	g/cm^3^
Young’s modulus	413	GPa
Poisson’s ratio	0.22	--
Thermal conductivity 20 °C	35	W/ m °K
Melting point	2273	°K

**Table 2:** Input parameters of RUM and their levels

RUM Input Parameters	Levels
Cutting speed, rpm	2000, 4500, 7000
Feed rate, mm/min	1, 2, 3
Depth of cut, mm	0.05, 0.1, 0.15
Vibration amplitude, µm	5, 10, 15
Vibration frequency, kHz	20, 25, 30
Constant parameters	
Tool type	Nickel bonded diamond
Coolant	Grindex 10
Coolant to distilled water ratio	1:10 (by volume)
Coolant pressure	4 bar

**Table 3:** Experimental results

Exp. #	RUM Input Parameters					Responses		
	Cutting Speed (S), rpm	Feed Rate (FR), mm/min	Depth of Cut (Doc), mm	Frequency (F), kHz	Amplitude (A), µm	Ra (µm)	EC (µm)	MRR (mm^3^/min)
1	7000	2	0.15	30	5	0.24	11.7	0.444
2	4500	3	0.1	25	10	0.35	12.2	0.441
3	4500	3	0.1	25	10	0.28	12.6	0.439
4	4500	4	0.1	25	10	0.31	12.1	0.587
5	2000	3	0.1	25	10	0.42	14.4	0.446
6	7000	4	0.15	20	5	0.36	14.6	0.850
7	4500	3	0.15	25	10	0.33	12.4	0.655
8	7000	4	0.05	30	5	0.29	9.2	0.298
9	2000	2	0.15	30	15	0.30	10.1	0.439
10	4500	3	0.1	25	10	0.32	13.5	0.443
11	7000	2	0.05	20	5	0.30	8.4	0.149
12	2000	2	0.05	20	15	0.32	13	0.150
13	4500	3	0.1	25	10	0.36	11.2	0.443
14	2000	2	0.15	20	5	0.38	14.9	0.439
15	4500	3	0.1	25	15	0.29	11.1	0.448
16	7000	2	0.15	20	15	0.25	13.7	0.447
17	4500	3	0.1	25	5	0.35	12.1	0.439
18	4500	3	0.1	20	10	0.37	12.7	0.444
19	2000	2	0.05	30	5	0.28	10.7	0.150
20	4500	2	0.1	25	10	0.35	11.5	0.298
21	2000	4	0.15	30	5	0.49	12.3	0.871
22	4500	3	0.1	25	10	0.23	9.7	0.444
23	7000	4	0.05	20	15	0.33	14.6	0.297
24	7000	3	0.1	25	10	0.26	10.9	0.444
25	2000	4	0.05	30	15	0.32	13.2	0.298
26	4500	3	0.1	25	10	0.30	13.5	0.443
27	4500	3	0.1	30	10	0.29	12.9	0.444
28	7000	4	0.15	30	15	0.32	12.8	0.871
29	2000	4	0.05	20	5	0.38	13.9	0.298
30	2000	4	0.15	20	15	0.42	17.3	0.885
31	7000	2	0.05	30	15	0.34	9.6	0.150
32	4500	3	0.05	25	10	0.29	11.4	0.223

**Table 4:** ANOVA results of Ra

Source	DF	Adj SS	Adj MS	F-Value	*p*-Value	% Contribution
Model	10	0.090733	0.009073	12.99	0.000 *	
Linear	5	0.054833	0.010967	15.71	0.000 *	
Cutting Speed (A)	1	0.008450	0.008450	12.10	0.002 *	9.2
Feed Rate (B)	1	0.007200	0.007200	10.31	0.004 *	7.9
Depth of Cut (C)	1	0.022756	0.022756	32.59	0.000 *	24.9
Frequency (D)	1	0.004672	0.004672	6.69	0.017 *	5.1
Amplitude (E)	1	0.011756	0.011756	16.84	0.001 *	12.9
Two-Way Interaction	5	0.035900	0.007180	10.28	0.000 *	
Cutting Speed × Frequency	1	0.003025	0.003025	4.33	0.049 *	3.3
Cutting Speed × Amplitude	1	0.009025	0.009025	12.92	0.002 *	9.9
Feed Rate × Frequency	1	0.004225	0.004225	6.05	0.023 *	4.6
Feed Rate × Amplitude	1	0.013225	0.013225	18.94	0.000 *	14.5
Frequency × Amplitude	1	0.006400	0.006400	9.17	0.006 *	7
Error	21	0.014664	0.000698			
Lack-of-Fit	16	0.011180	0.000699	1.00	0.549 **	
Pure Error	5	0.003483	0.000697			0.7
Total	31	0.105397				
R^2^	94.45%					

* Significant. ** Non significant.

**Table 5:** ANOVA results of EC

Source	DF	Adj SS	Adj MS	F-Value	*p*-Value	% Contribution
Model	10	69.3181	6.9318	13.63	0.000 *	
Linear	5	36.9406	7.3881	14.53	0.000 *	
Cutting Speed (A)	1	7.4756	7.4756	14.70	0.001 *	10.78
Feed Rate (B)	1	5.1200	5.1200	10.07	0.005 *	7.39
Depth of Cut (c)	1	12.8356	12.8356	25.25	0.000 *	18.5
Frequency (D)	1	4.9089	4.9089	9.66	0.005 *	7.1
Amplitude (E)	1	6.6006	6.6006	12.98	0.002 *	9.5
Two-Way Interaction	5	32.3775	6.4755	12.74	0.000 *	
Cutting Speed × Depth of Cut	1	5.5225	5.5225	10.86	0.003 *	8
Cutting Speed × Frequency	1	16.4025	16.4025	32.26	0.000 *	23.7
Feed Rate × Depth of Cut	1	2.7225	2.7225	5.35	0.031 *	3.9
Feed Rate × Amplitude	1	4.8400	4.8400	9.52	0.006 *	7
Depth of Cut × Amplitude	1	2.8900	2.8900	5.68	0.027 *	4.1
Error	21	10.6769	0.5084			
Lack-of-Fit	16	9.6886	0.6055	3.06	0.110 **	
Pure Error	5	0.9883	0.1977			0.03
Total	31	79.9950				
R^2^	92.54%					

* Significant. ** Non significant.

**Table 6:** ANOVA results of MRR

Source	DF	Adj SS	Adj MS	F-Value	*p*-Value	% Contribution
Model	13	1.29147	0.099344	28435.72	0.000 *	
Linear	5	1.21286	0.242572	69432.58	0.000 *	
Cutting Speed (A)	1	0.00004	0.000037	10.47	0.005 *	0.002
Feed Rate (B)	1	0.37232	0.372315	106569.77	0.000 *	28.9
Depth of Cut (C)	1	0.84038	0.840377	240545.64	0.000 *	65.1
Frequency (D)	1	0.00000	0.000001	0.35	0.561 **	0
Amplitude (E)	1	0.00013	0.000128	36.68	0.000 *	0.01
Square	1	0.00010	0.000100	28.49	0.000 *	
Depth of Cut × Depth of Cut	1	0.00010	0.000100	28.49	0.000 *	0.007
Two-Way Interaction	7	0.07851	0.011216	3210.43	0.000 *	
Cutting Speed × Feed Rate	1	0.00015	0.000151	43.23	0.000 *	0.01
Cutting Speed × Depth of Cut	1	0.00003	0.000025	7.25	0.015 *	0.001
Cutting Speed × Frequency	1	0.00006	0.000061	17.41	0.001 *	0.004
Feed Rate × Depth of Cut	1	0.07799	0.077989	22323.31	0.000 *	6.04
Feed Rate × Amplitude	1	0.00006	0.000059	16.96	0.001 *	0.004
Depth of Cut × Amplitude	1	0.00009	0.000093	26.52	0.000 *	0.007
Frequency × Amplitude	1	0.00013	0.000134	38.34	0.000 *	0.01
Error	18	0.00006	0.000003			
Lack-of-Fit	13	0.00005	0.000003	1.01	0.541 **	
Pure Error	5	0.00002	0.000003			0.001
Total	31	1.29153				
R^2^	99.9%					

* Significant. ** Non significant.

**Table 7:** Comparison of the chemical composition of as-received material and the machined micro-pockets.

Selected Region for EDS Analysis	Elements	Weight %	Atomic %
As-received material	Al	51.37	40.55
	O	48.63	59.45
Micro-pockets	Al	53.32	42.40
	O	46.68	57.60

**Table 8:** Boundaries of RUM parameters for Ra, EC, and MRR

Parameter	Target	Lower Bound	Upper Bound	Weight	Importance	
Cutting speed (rpm)	In range	2000	7000	1	1	
Feed rate (mm/min)	In range	2	4	1	1	
Depth of Cut (mm)	In range	0.05	0.15	1	1	
Frequency (kHz)	In range	20	30	1	1	
Amplitude (µm)	In range	5	15	1	1	
**Responses**						**Composite Desirability**
Ra	Minimize	0.23	0.49	1	3	1
EC	Minimize	8.40	17.3	1	3	1
MRR	Maximize	0.149	0.8852	1	3	1

**Table 9:** Results of single response optimization

Optimization Condition					Responses			
Cutting Speed (rpm)	Feed Rate (mm/min)	Depth of Cut (mm)	Frequency (kHz)	Amplitude (µm)	Responses	Optimal	Predicted	Error%
7000	2	0.05	30	5	Ra (µm)	0.164	0.168	2.3
2000	2	0.05	30	5	EC (µm)	8.963	8.953	0
2000	4	0.15	20	15	MRR (mm^3^/min)	0.885	0.879	−0.67

**Table 10:** Experimental results for Ra, EC, and MRR at optimized settings and confirmatory results

Optimization Condition					Responses			
Cutting Speed (rpm)	Feed Rate (mm/min)	Depth of Cut (mm)	Frequency (kHz)	Amplitude (µm)	Responses	Optimal	Predicted	Error%
6787	3.52	0.13	21.8	13.7	Ra (µm)	0.301	0.315	−4.44
					EC (µm)	12.450	13.04	−4.50
					MRR (mm^3^/min)	0.873	0.886	1.46
